# Content of nitric oxide and glycative compounds in cured meat products-Negative impact upon health

**DOI:** 10.1051/bmdcn/2018080210

**Published:** 2018-05-28

**Authors:** Ya-chen Yang, Zhi-hong Wang, Mei-chin Yin

**Affiliations:** a Department of Food Nutrition and Health Biotechnology, Asia University Taichung 413 Taiwan; b Department of Medical Research, China Medical University Hospital, China Medical University Taichung 404 Taiwan

**Keywords:** Cured meat, Nitric oxide, 3-Nitrotyrosine, Pentosidine, Carboxymethyllysine

## Abstract

The content of nitric oxide (NO), 3-nitrotyrosine, advanced glycation endproducts (AGEs) and trans fatty acids (TFAs) in 16 kinds of cured meat products was examined. Results showed that NO and 3-nitrotyrosine levels were in the range of non-detectable to 4.6 μM/mg protein, and non-detectable to 0.49 nmol/mg protein, respectively. Carboxymethyllysine could be detected in 13 kinds of cured meat products; its content was in the range of 48-306 μg/100 g meat. Pentosidine was found in 14 kinds of meat products, in the range of 109-631 μg/100 g meat. Furosine was presented in all test meat samples, in the range of 156-676 μg/100 g meat. Palmitelaidic acid was found in 3 kinds of meat product, and the content was in the range of 0.59-0.71%. Vaccenic acid was presented in 9 kinds of meat products; its content was in the range of 0.89-1.47%. Elaidic acid was detected in 5 kinds of meat products, and the content was in the range of 0.67-1.21%. Because NO, 3-nitrotyrosine and AGEs might have adverse impact upon health, people with certain healthy conditions should carefully consider the frequency and amount of consumption for these cured meat products.

## 1. Introduction

Many cured meat products such as sausage, bacon, hot dog and ham are often served in western diets. Nitrites are used as preservatives, and contribute to the typical red curing color, anti-microbial protection and special flavor in cured meat products [1]. It is reported that high intake of cured meat facilitated the progression of respiratory disorders because nitrites induced nitrosative stress in airway system [2]. Honikel [3] indicated that nitric oxide (NO), a free radical with toxic property, could be formed in cured meat from nitrites through auto-decomposition. It is reported that excessive NO may cause endothelial dysfunctions *via* increasing oxidative and inflammatory stress, which consequently promotes the progression of Parkinson's disease or muscular dystrophy [4, 5]. Thus, it is meaningful to examine the content of NO and other nitro-oxidants such as 3-nitrotyrosine in cured meat in order to further understand the impact of cured meat upon health.

Although each brand or company has its own recipe for cured meat preparation, sugar is commonly added for making cured meat products. Thus, it is highly possible that cured meat products are dietary advanced glycation endproducts (AGEs) source because sugar could react with amino acid(s) through maillard reaction. Carboxymethyllysine (CML), pentosidine and furosine are common AGEs found in many foods such as heated milk and cookies [6, 7]. It is reported that dietary AGEs contribute to increase circulating AGE level, and favor the development of glycation associated diseases such as diabetes and Alzheimer's disease [8]. So far, less attention was paid to the content of AGEs in cured meat products. In addition, the occurrence of trans fatty acids (TFAs) in beef and many processed foods has been reported [9, 10]. The common trans fatty acids occurred in foods are palmitelaidic acid (PA, C16:1n-7t), vaccenic acid (VA, C18:1n-7t), elaidic acid (EA, C18:1n-9t) and linoelaidic acid (LA, C18:2n- 6t,9t) [11]. These dietary TFAs raise the risk of atherosclerosis, coronary insufficiency, metabolic diseases and myocardial infarction [12]. Thus, it is curious about the content of TFAs in cured meat products.

In our present study, the content of NO, 3-nitrotyrosine, AGEs and TFAs in cured meat products was examined. Microbiological analysis for these meat products was also processed in order to exclude the influence of microorganisms upon these examined factors. The results of this study could provide some information for consumers to consider whether the intake of cured meat products should be limited in order to prevent the development or progression of associated diseases.

## 2. Materials and Methods

### 2.1. Meat sample

There were 16 kinds of cured meat products used in this study. They were: 1. Hungarian salami, 2. Smoked sausage with cheddar chess, 3. Smoked brats: cured, cooked & smoked bratwurst, 4. Ham off the bone, 5. Pork hot dog, 6. Bulgogi beef jerky, 7. Pork strips, 8. Prosciutto, 9. Smoked sliced beef, 10. Peppered beef, 11. Smoked ham, 12. Roast pork, 13. Bacon, 14. Frankfurter style sausage, 15. Bavarian style sausage, 16. Loin ham. These meat products were purchased from local supermarkets or wholesalers in Taichung City, Taiwan, in September 2015-August, 2016. Meat products #1-#8 were imported from other countries including USA, Australia or Italian. Meat products #9-#16 were products of Taiwan. For each kind of cured meat product, 7 samples with 7 different lot numbers were collected. For each lot, 2 meat samples (with the same lot number) were purchased. The total of 224 meat samples was used in this study. The analyses for all meat samples were processed before they were expired.

### 2.2. Microbiological analysis

Ten g meat sample was mixed with 90 *ml* sterile 0.1% peptone water, and followed by homogenizing in a stomacher. After serially dilutions by transferring 1 *ml* to 9 *ml* of peptone water, 0.1 *ml* sample was spread on Plate Count Agar for total viable aerobic count (TVAC) after 48 hr incubation at 30°C; on Eosin Methylene Blue Agar for coliforms recover after 48 hr incubation at 37°C; on Potato Dextrose Agar for recovering yeast and molds after 72 hr incubation at 25°C. These agars were obtained from Difco Laboratory (Detroit, MI, USA).

### 2.3. Assays for NO and 3-nitrotyrosine

Meat sample at 50 g was homogenized in 50 *ml* distilled water. After filtrating through a Whatman No.1 filter paper, the filtrate was collected. NO level (μM/mg protein) was analyzed by measuring nitrite formation. In brief, 100 μl sample homogenate was reacted with nitrate reductase, NADPH and FAD. After incubation in the dark for 1 hr at 37°C, this mixture was centrifuged at 6,000 xg. The supernatant was collected and mixed with Griess reagent. The absorbance at 540 nm was measured, and compared with a sodium nitrite standard curve. 3-Nitrotyrosine level (nmol/mg protein) was assayed by an ELISA kit (Northwest Life Science Specialties, Vancouver, WA, USA). Protein content of homogenate was determined by an assay kit (Pierce Biotechnology Inc., Rockford, IL, USA), and the standard was bovine serum albumin.

### 2.4. Measurement of CML, pentosidine and furosine

CML level was determined by the method described in Niquet-Leridon and Tessier [13]. In brief, meat sample at 50 g was chopped and mixed with NaBH_4_ in borate buffer (pH 9.1) for reduction. Trichloroacetic acid (TCA) at 20% was added to precipitate proteins, and followed by centrifugation for 10 min at 2,000 xg. After washing with 1 *ml* 10% TCA, the collected pellet was placed in screw-cap tubes and acid hydrolyzed by 500 μl HCl (6 M) for 16 hr at 110°C. CML was quantified by using a Surveyor HPLC system, in which a Finnigan LTQ ion trap mass spectrometer (Thermo-Fisher Scientific, Courtaboeuf, France) was coupled. A hypercarb column (100 × 2.1 mm, 5 μm) was used for separation. Pentosidine was measured by the HPLC method described in Miyata *et al.* [14], in which HPLC was equipped with a fluorescence detector (RF-10A Shimadzu, Kyoto, Japan) and a reverse phase C18 column (4.6 mm id; 25 cm od) purchased from Supelco Co. (Bellefonte, PA, USA). Wavelengths of excitation and emission were 335 and 385 nm, respectively. Furosine level was analyzed by the method described in Delgado *et al.* [15]. Briefly, 10 *ml* homogenate was hydrolyzed by using 3 *ml* HCL (7.95 M) at 110°C for 23 hr in screw-cap tubes. After hydrolysates were cooled down and centrifuged at 14,000 xg for 10 min, the supernatant was collected to run through a Sep-Pak C18 cartridge (Millipore, Milfold, MA, USA). This cartridge was pre-wetted by deionized water and methanol at 10 *ml* and 5 *ml,* respectively. After eluted by 3 *ml* HCl (3 M), the sample was redissolved in 1 *ml* solution consisted of distilled water, acetonitrile and formic acid (95:5:0.2, v/v/v). The mobile phase was 5 mM sodium heptane sulphonate containing 0.2% formic acid and 20% acetonitrile.

### 2.5. Determination of TFAs

The content of TFAs, PA, VA, EA or LA, was analyzed by a HP5890 gas chromatography (Hewlett Packard, Palo Alto, CA, USA) equipped with a flame ionization detector. A 30-m Omegawax capillary column (Supelco Chromatography Products, Bellefonte, PA, USA) was applied for TFAs measurement. Content of each fatty acid was quantified by measuring the areas under each identified peak. Results are shown as % of total fatty acids.

### 2.6. Statistical analysis

For each kind of cure meat product, seven different meat samples were used to determine the effect of each measurement (n = 7). Data are reported as means ± standard deviation (SD). Statistical analyses were processed by one-way analysis of variance. Least Significance Difference Test was used to determine the differences among means, and significance was considered as *P* value < 0.05.

## 3. Results

### 3.1. Level of microorganisms, NO and 3-nitrotyrosine

The level (log_10_ cfu/g) of TVAC, coliforms, and yeast and molds in test meat products is presented in Table 1. TVAC could be detected in all meat products, in the range of 0.6-2.5 log_10_ cfu/g. Coliforms were found in 7 kinds of meat samples, in the range of 0.5-1.3 log_10_ cfu/g. Yeast and molds were detected in 4 kinds of meat products, in the range of 0.3-0.7 log_10_ cfu/g. As shown in Table 2, NO was detectable in 12 kinds of meat samples, and its level was in the range of 0.8-4.6 μM/mg protein. 3-Nitrotyrosine was found in 9 kinds of meat samples, in the range of 0.21-0.49 nmol/mg protein. Bulgogi beef jerky, pork strips and bavarian style sausage, #6, #7 and #15, had greater NO and 3-nitrotyrosine levels than other meat samples (P < 0.05).

**Table 1:** Level (log_10_ cfu/g) of TVAC, coliforms and yeast and molds (Y&M) of cure meat products. Data are mean ± SD, n = 7.

Meat products #	TVAC	coliforms	Y & M
1	1.6 ± 0.3^b^	1.0 ± 0.5^b^	*,^a^
2	1.3 ± 0.2^b^	*,^a^	*,^a^
3	1.9 ± 0.4^b^	0.9 ± 0.2^b^	*,^a^
4	2.2 ± 0.5^c^	0.6 ± 0.4^b^	0.5 ± 0.4^b^
5	1.7 ± 0.3^b^	*,^a^	*,^a^
6	1.8 ± 0.6^b^	1.3 ± 0.6^b^	0.6 ± 0.2^b^
7	0.9 ± 0.2^a^	*,^a^	*,^a^
8	2.3 ± 0.4^c^	*,^a^	*,^a^
9	1.5 ± 0.3^b^	*,^a^	*,^a^
10	0.8 ± 0.1^a^	*,^a^	*,^a^
11	1.3 ± 0.8^b^	0.8 ± 0.4^b^	0.7 ± 0.3^b^
12	2.5 ± 0.7^c^	*,^a^	*,^a^
13	0.6 ± 0.3^a^	0.7 ± 0.6^b^	*,^a^
14	1.4 ± 0.2^b^	*,^a^	*,^a^
15	0.8 ± 0.4^a^	0.5 ± 0.2^b^	0.3 ± 0.2^b^
16	1.3 ± 0.6^b^	*,a	*,a

* Means too low to be detected.

a Values among means in a column without a common letter differ, *P* < 0.05.

b Values among means in a column without a common letter differ, *P* < 0.05.

c Values among means in a column without a common letter differ, *P* < 0.05.

**Table 2:** Level of NO (mM/mg protein) and 3-nitrotyrosine (nmol/mg protein) in cured meat products. Data are mean ± SD, n = 7.

Meat products #	NO	3-nitrotyrosine
1	3.2 ± 0.5^d^	0.31±0.09^b^
2	2.3 ± 0.3^c^	0.46±0.07^c^
3	*,^a^	*,^a^
4	2.7 ± 0.4^c^	0.44±0.08^c^
5	*,^a^	*,^a^
6	4.4 ± 0.2^e^	0.49±0.05^c^
7	4.6 ± 0.7^e^	0.42±0.06^c^
8	2.9 ± 0.5^c^	0.21±0.03^b^
9	0.8 ± 0.4^b^	*,^a^
10	1.5 ± 0.6^b^	*,^a^
11	2.1 ± 1.0^c^	0.27±0.04^b^
12	1.3 ± 0.7^b^	0.24±0.06^b^
13	*,^a^	*,^a^
14	1.0 ± 0.9^b^	*,^a^
15	4.2 ± 0.6^e^	0.45±0.07^c^
16	*,^a^	*,^a^

* Means too low to be detected.

a Values among means in a column without a common letter differ, *P* < 0.05.

b Values among means in a column without a common letter differ, *P* < 0.05.

c Values among means in a column without a common letter differ, *P* < 0.05.

d Values among means in a column without a common letter differ, *P* < 0.05.

e Values among means in a column without a common letter differ, *P* < 0.05.

### 3.2. AGEs content

As shown in Table 3, CML could be detected in 13 kinds of cured meat products, in the range of 48-306 μg/100 g meat. Pentosidine was found in 14 kinds of meat products, in the range of 109-631 μg/100 g meat. Furosine was presented in all test meat samples, in the range of 156-676 μg/100 g meat. Bulgogi beef jerky, pork strips and prosciutto, items #6-#8, had greater total AGEs level than other meat samples *(P* < 0.05).

**Table 3:** Content (mg/100 g sample) of CML, pentosidine and furosine in cured meat products. Data are mean ± SD, n = 7.

Meat products #	CML	pentosidine	furosine	Total AGEs
1	66 ± 12^b^	265 ± 70^c^	482 ± 83^d^	813 ± 76^d^
2	48 ± 9^b^	130 ± 44^b^	182 ± 38^a^	360 ± 35^a^
3	59 ± 10^b^	168 ± 53^b^	216 ± 30^b^	443 ± 42^b^
4	135 ± 21^c^	147 ± 29^b^	331 ± 58^c^	613 ± 54^c^
5	*,^a^	127 ± 35^b^	191 ± 15^a^	318 ± 29^a^
6	154 ± 26^c^	549 ± 84^e^	676 ± 62^e^	1379 ± 80^e^
7	193±23^d^	631± 62^e^	553 ± 41^e^	1351 ± 70^e^
8	306 ± 34^e^	551 ± 38^e^	431 ± 29^d^	1288 ± 47^e^
9	116 ± 19^c^	244 ± 37^c^	360 ± 51^c^	720 ± 43^d^
10	*,^a^	109 ± 30^b^	156 ± 18^a^	265 ± 32^a^
11	129 ± 20^c^	342 ± 47^d^	376 ± 63^c^	847± 51^d^
12	*,^a^	117 ± 25^b^	381 ± 56^c^	498 ± 47^b^
13	71 ± 13^b^	*,^a^	247 ± 39^b^	318 ± 27^a^
14	148 ± 25^c^	208 ± 42^c^	290 ± 55^b^	646 ± 39^c^
15	207 ± 36^d^	150 ± 44^b^	263 ± 60^b^	620 ± 48^c^
16	52 ± 6^b^	*,^a^	198 ± 56^a^	250 ± 33^a^

* Means too low to be detected.

a Values among means in a column without a common letter differ, *P* < 0.05.

b Values among means in a column without a common letter differ, *P* < 0.05.

c Values among means in a column without a common letter differ, *P* < 0.05.

d Values among means in a column without a common letter differ, *P* < 0.05.

e Values among means in a column without a common letter differ, *P* < 0.05.

### 3.3. TFAs content

TFAs content in meat products is shown in Table 4. PA was found in 3 kinds of meat product, and its content was in the range of 0.59-0.71%. VA was presented in 9 kinds of meat products; its content was in the range of 0.89-1.47%. EA was detected in 5 kinds of meat products, and the content was in the range of 0.67-1.21%. LA was not detectable in all test mat samples. Smoked sausage with cheddar chess, bulgogi beef jerky and pork strips, #2, #6 and #7, had greater total TFAs than other meat samples (P < 0.05).

**Table 4:** Content (% of total fatty acids) of four trans fatty acids in cured meat products. Data are mean ± SD, n = 7.

Meat products #	PA	VA	EA	LA	Total TFAs
1	*,^a^	1.24 ± 0.17^c^	0.96 ± 0.29^b^	*,^a^	2.20 ± 0.23^d^
2	*,^a^	1.47 ± 0.36^c^	1.21 ± 0.31^b^	*,^a^	2.68 ± 0.33^e^
3	0.71 ± 0.1^b^	1.14 ± 0.18^b^	*,^a^	*,^a^	1.85 ± 0.14^c^
4	*,^a^	*,^a^	*,^a^	*,^a^	*,^a^
5	*,^a^	*,^a^	*,^a^	*,^a^	*,^a^
6	0.62 ± 0.21^b^	1.40 ± 0.35^c^	0.81 ± 0.28^b^	*,^a^	2.83 ± 0.27^e^
7	*,^a^	1.29 ± 0.11^c^	1.13 ± 0.32^b^	*,^a^	2.42 ± 0.18^e^
8	*,^a^	0.92 ± 0.24^b^	0.67 ± 0.27^b^	*,^a^	1.59 ± 0.25^d^
9	*,^a^	*,^a^	*,^a^	*,^a^	*,^a^
10	*,^a^	*,^a^	*,^a^	*,^a^	*,^a^
11	*,^a^	*,^a^	*,^a^	*,^a^	*,^a^
12	0.59 ± 0.13^b^	1.08 ± 0.19^b^	*,^a^	*,^a^	1.67 ± 0.16^c^
13	*,^a^	1.36 ± 0.34^c^	*,^a^	*,^a^	1.36 ± 0.34^d^
14	*,^a^	*,^a^	*,^a^	*,^a^	*,^a^
15	*,^a^	0.89 ± 0.11^b^	*,^a^	*,^a^	0.89 ± 0.11^c^
16	*,^a^	*,^a^	*,^a^	*,^a^	*,^a^

* Means too low to be detected.

a Values among means in a column without a common letter differ, *P* < 0.05.

b Values among means in a column without a common letter differ, *P* < 0.05.

c Values among means in a column without a common letter differ, *P* < 0.05.

d Values among means in a column without a common letter differ, *P* < 0.05.

e Values among means in a column without a common letter differ, *P* < 0.05.

## 4. Discussion

In our present study, the microbiological data revealed that the 16 kinds of cured meat products were safe based on the low counts in aerobic bacteria, coliforms, yeast and molds. Thus, the influence of microorganisms upon detected NO, 3-nitrotyrosine, AGEs and TFAs in these cured meat products could be excluded.

The adverse effects of cured meats upon respiratory system in patients has been reported [16], and those authors indicated that high nitrite content of cured meat raised nitrosative stress associated inflammation in airways. Cooke and Ghebremariam [17] reported that dietary nitrites could be absorbed in the gas trointestinal tract, which in turn increased circulating level of NO. Our present study further found that these cured meat products had substantial content of NO and 3-nitrotyrosine. Obviously, not only nitrites but also nitro-oxidants such as NO and/ or 3-nitrotyrosine could be obtained through the intake of these cured meat products, which subsequently could enhance oxidative and inflammatory stresses in circulation. It is possible that NO was formed due to the degradation of nitrites or nitrates used for meat curing process; and 3-nitrotyrosine was formed due to protein nitration in those cured meat products. Although NO could protect cardiovascular system *via* acting as a vasorelaxant agent and modulating vascular tone [18], NO at higher level was a toxic substance and impaired health. The study of Nagy *et al.* [19] revealed that NO caused T lymphocyte dysfunction in auto-immune diseases such as systemic lupus erythematosus and rheumatoid arthritis. In addition, plasma 3-nitrotyrosine level is a biomarker of arthritis [20]. Thus, the increased dietary intake of NO and 3-nitrotyrosine might cause adverse impact upon respiratory and immune systems. Our present study indicated that cured meats are food sources of NO and 3-nitrotyrosine. Apparently, the intake of these cured meat products for patients under certain pathological conditions should be restricted.

CML, pentosidine and furosine could be formed by the reaction of lysine or arginine with sugars or dicarbonyl compounds [7, 21]. Thus, the presence of these AGEs in foods suggested that the bio-available amino acids such as lysine and arginine were reduced. For cured meat preparation, heating process is often used and some ingredients such as sugar, salt and emulsifying agent are commonly added. These factors lead to the formation of AGEs in meat products. Thus, it seems reasonable to observe the presence of AGEs in these cured meat products. It is reported that dietary AGEs intake increased circulating AGEs levels [22]. Consequently, AGEs in circulation respond with their receptors, also called as RAGE. The engagement of AGE with RAGE could activate oxidation and inflammation associated signaling pathways, and subsequently stimulate the production of oxidants and inflammatory cytokines [23], and finally promote the development and progression of glycation associated diseases including diabetes, renal failure, aging and Alzheimer's disease [8]. Our present study found that cured meat products are food source of AGEs. Thus, people with high risk of these glycative diseases should reduce the consumption of cured meat products.

VA, the predominant ruminant-derived TFA, has been considered as a good TFA because dietary supplement of VA could attenuate dyslipidemia, hepatic steatosis, and inflammation [24, 25]. In our present study, VA could be detected in 9 kinds of cured meat products. The presence of VA in those meat products might provide nutritional benefits for consumers. On the other hand, PA, EA and LA are considered as bad TFAs because EA and LA promoted inflammatory response in endothelial cells; dietary PA and EA were associated with higher mortality of cardiovascular diseases and cancer [26, 27]. The studies of Dannenberger *et al.* [28] and Alfaia *et al.* [29] revealed that fatty acid composition of beef muscle or by-products from bovines could be affected by diets. Thus, the detected TFAs in these cured meat products as we observed may be from their raw materials, beef or pork. Another possibility is that heat treatment or additives used for making these cured met products modified the fatty acid profiles. In our present study, PA and EA were detectable only in few meat samples and their content were low, and LA was not found in all test meat samples. Therefore, it is hard to conclude that cured meat products are food sources of bad TFAs.

## 5. Conclusion

Cured meat products are rich in proteins and easily served for meal. However, our present study found that 16 kinds of cured meat products contained nitric oxide, 3-nitrotyrosine and several glycative compounds. These components might have adverse impact upon human health. These findings suggest that the people with certain healthy conditions should carefully consider the frequency and amount of consumption for these cured meat products.

## Conflicts of interest statement

There was no Conflict of Interest regarding this manuscript.
